# Supplementary material for: Identification of photoperiod-induced specific miRNAs in the adrenal glands of Sunite sheep (Ovis aries)
Source: Front Vet Sci. 2022 Jul 22;9:888207. doi: 10.3389/fvets.2022.888207 (PMC9354845; doi:10.3389/fvets.2022.888207)
Supplement: Supplementary file 1 [file Data_Sheet_1.ZIP › Supplementary file/miRDeep2 file.zip!/miRDeep2 file/Know_Structure/SPLP42A3/oar-mir-380.pdf]

miRBase precursor : oar-mir-380
Total read count : 3192
oar-miR-380-5p read count : 21
oar-miR-380-3p read count : 3171
remaining reads : 0

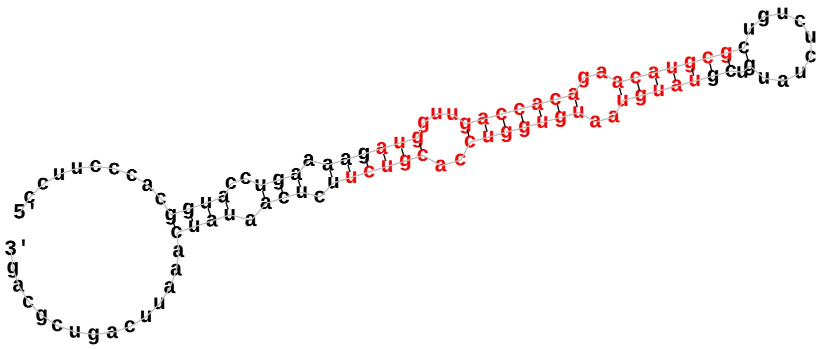

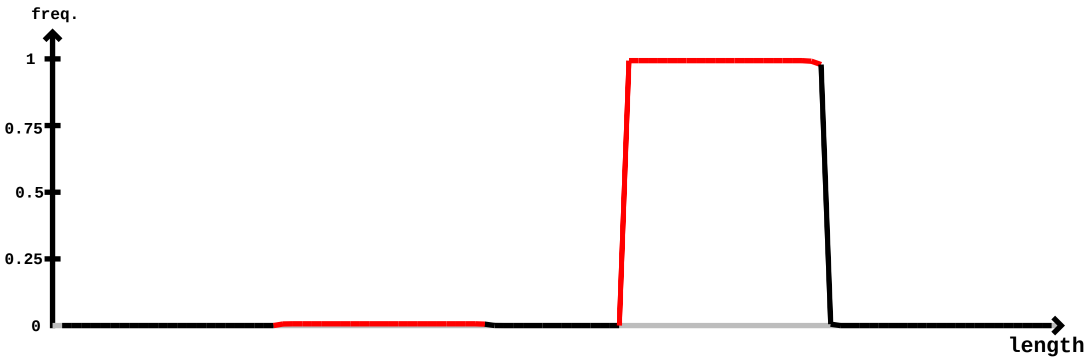

oar-miR-380-5p

oar-miR-380-3p

```
5'- ccuucccacgguaccugaaaagaugguugaccacagaacaugcgcugucucuaugucguauguaaugugguccacgucuucucaauaucaaauucagucgcag   -3'   exp
    .........((((..(((.((((((...(((((((..((((((((.........).)))))))..)))))))..)))))).))).)))).............. reads   mm   sample
    ......................augguugaccacagaacaugc............................................................ 4       0    seq
    ......................augguugaccacagaacaugcg........................................................... 14      0    seq
    ......................augguugaccacagaacaugcgc.......................................................... 1       0    seq
    .......................ugguugaccacagaacaugcg........................................................... 2       0    seq
    ..........................................................uauguaaugugguccacgu.......................... 7       0    seq
    ..........................................................uauguaaugugguccacguc......................... 40      0    seq
    ..........................................................uauguaaugugguccacgucu........................ 3107    0    seq
    ..........................................................uauguaaugugguccacgucuu....................... 17      0    seq
```
